# Discriminating Pathological and Non-pathological Internet Gamers Using Sparse Neuroanatomical Features

**DOI:** 10.3389/fpsyt.2018.00291

**Published:** 2018-06-29

**Authors:** Chang-hyun Park, Ji-Won Chun, Hyun Cho, Dai-Jin Kim

**Affiliations:** ^1^Department of Psychiatry, Seoul St. Mary's Hospital, College of Medicine, Catholic University of Korea, Seoul, South Korea; ^2^Department of Psychology, Korea University, Seoul, South Korea

**Keywords:** internet gaming disorder, diagnostic classification, structural MRI, diffusion-weighted MRI, regularized regression

## Abstract

Internet gaming disorder (IGD) is often diagnosed on the basis of nine underlying criteria from the latest version of the Diagnostic and Statistical Manual of Mental Disorders (DSM-5). Here, we examined whether such symptom-based categorization could be translated into computation-based classification. Structural MRI (sMRI) and diffusion-weighted MRI (dMRI) data were acquired in 38 gamers diagnosed with IGD, 68 normal gamers diagnosed as not having IGD, and 37 healthy non-gamers. We generated 108 features of gray matter (GM) and white matter (WM) structure from the MRI data. When regularized logistic regression was applied to the 108 neuroanatomical features to select important ones for the distinction between the groups, the disordered and normal gamers were represented in terms of 43 and 21 features, respectively, in relation to the healthy non-gamers, whereas the disordered gamers were represented in terms of 11 features in relation to the normal gamers. In support vector machines (SVM) using the sparse neuroanatomical features as predictors, the disordered and normal gamers were discriminated successfully, with accuracy exceeding 98%, from the healthy non-gamers, but the classification between the disordered and normal gamers was relatively challenging. These findings suggest that pathological and non-pathological gamers as categorized with the criteria from the DSM-5 could be represented by sparse neuroanatomical features, especially in the context of discriminating those from non-gaming healthy individuals.

## Introduction

Although having been suggested as pathological addiction for decades (1), it is only recently that Internet gaming disorder (IGD) was listed in the Diagnostic and Statistical Manual of Mental Disorders (DSM). The fifth edition of the DSM (DSM-5) (2) identified IGD as a condition for further study and provided nine criteria for diagnosing it. In symptom-based categorization using the nine-item IGD scale (IGDS) proposed in the DSM−5, a threshold of experiencing five or more criteria was applied to the diagnosis of IGD. Although this cut-point may adequately differentiate gamers suffering significant clinical impairment (3), the dichotomous nature of IGDS items inevitably involves diagnostic oversimplification or vagueness.

Besides symptoms, a variety of IGD-related dysfunctions are commonly observed, not least neuroanatomical changes. Indeed, a substantial body of work has shown that IGD is associated with structural alterations in the brain: shrinkage of gray matter (GM) volume (4–6), reduction in cortical thickness (7), and loss of white matter (WM) integrity (8, 9) have been typically demonstrated. These neuroanatomical changes related to IGD suggest that such brain imaging parameters can serve as biomarkers to distinguish individuals with IGD from other individuals. That is, the diagnosis of IGD may be made through computational manipulation of neuroanatomical biomarkers, rather than through symptom-based categorization based on the DSM-5. These attempts may be in line with efforts to move beyond descriptive diagnosis by employing computational approaches to psychiatry (10), specifically data-driven approaches based on machine learning (ML) to tackle the diagnosis of mental illness (11).

In this study, we searched for a link between symptom-based categorization on the basis of the IGDS and computation-based classification by using neuroanatomical biomarkers in the diagnosis of IGD. Because some GM and WM components of the brain would be likely to include redundant or irrelevant information for diagnostic classification, we sought to select sparse neuroanatomical features by employing regularized regression. We hypothesized that symptom-based categorization could be represented in terms of sparse neuroanatomical features that would compose classification models for the diagnosis of IGD. Pathological gamers diagnosed with IGD were thought to be more dissimilar from non-gaming healthy individuals than from gamers diagnosed as not having IGD, that is, non-pathological gamers; thus, pathological gamers could be characterized by a larger number of features compared with non-pathological gamers, in relation to non-gaming healthy individuals. In addition, we wanted to decide whether non-pathological gamers could be less distinguishable from pathological gamers or from non-gaming healthy individuals. Non-pathological gamers might be vaguely assumed to be close to non-gaming healthy individuals in terms of descriptive symptoms, but we thought that such a notion needs to be validated by means of computation-based classification.

## Materials and methods

### Participants

Among 237 participants playing Internet-based games, 106 individuals were selected by excluding those who exhibited a mismatch between the self-reported IGDS and a structured interview with a clinical psychologist in the diagnosis of IGD or had missed or severely distorted brain imaging data. On the basis of the IGDS, 38 individuals (27.66 ± 5.61 years; 13 females) who satisfied at least five IGDS items were labeled disordered gamers and 68 individuals (27.96 ± 6.41 years; 21 females) who satisfied at most one IGDS item were labeled normal gamers. Individuals who satisfied IGDS items between two and four were also excluded, because they may be discerned as another class between the disordered and normal gamers (12). In addition, 37 individuals (25.86 ± 4.10 years; 13 females) not playing Internet-based games were separately recruited, and they were labeled healthy non-gamers. The absence of comorbidities in all participants was confirmed. Written informed consent was obtained from all participants in accordance with the Declaration of Helsinki and its later amendments, and the study was approved by the Institutional Review Board at the Seoul St. Mary's Hospital, Seoul, Korea.

### Acquisition of MRI data

Structural MRI (sMRI) and diffusion-weighted MRI (dMRI) data were collected using a 3 T MAGNETOM Verio system (Siemens AG, Erlangen, Germany). The acquisition of sMRI data was conducted using a magnetization-prepared rapid gradient echo sequence: number of slices in the sagittal plane = 176, slice thickness = 1 mm, matrix size = 256 × 256, and in-plane resolution = 1 × 1 mm. For the acquisition of dMRI data, diffusion gradient encoding was performed in 30 directions with *b* = 1,000 s/mm^2^ and a single-shot echo-planar imaging sequence was used: number of slices in the axial plane = 75, slice thickness = 2 mm, matrix size = 114 × 114, and in-plane resolution = 2 × 2 mm.

### Processing of MRI data

Tools included in CAT12 (http://www.neuro.uni-jena.de/cat/) were used to process sMRI data. The brain volume image was segmented into different tissues, including GM, WM, and corticospinal fluid as well as spatially registered to a reference brain in the standard space. In voxel-based morphometry (VBM), voxel-wise GM volume was estimated by multiplying the probability of being GM by the volume of a voxel, and then those values were divided by the total intracranial volume to adjust for individual differences in head volume. In surface-based morphometry (SBM), cortical thickness was estimated using the projection-based thickness method (13).

### Processing of dMRI data

Tools included in FSL 5.0 (http://fsl.fmrib.ox.ac.uk/fsl/) were employed to process dMRI data. All images were realigned to the null image acquired with *b* = 0 s/mm^2^ to correct for eddy current-induced distortions and head motion. A diffusion tensor was modeled at each voxel within the brain, and diffusion tensor-derived parameters, including fractional anisotropy (FA), mean diffusivity (MD), axial diffusivity (AD), and radial diffusivity (RD), were computed; given three diffusivities along different axes of a diffusion tensor, FA was calculated as the square root of the sum of squares of diffusivity differences between the three axes, MD as the average diffusivity across the three axes, AD as the greatest diffusivity along the principal axis, and RD as the average of diffusivities along two minor axes. Using tract-based spatial statistics (TBSS) (14) implemented in FSL 5.0, the maps of diffusion tensor-derived parameters were spatially registered to a reference brain in the standard space, and they were then projected onto a WM tract skeleton.

### Feature generation

Two major steps for designing a classification model are feature generation and selection. We generated features from neuroanatomy, specifically the volume and thickness of a set of GM regions and the integrity and diffusivity of a set of WM tracts. After estimating GM volume and cortical thickness as voxel-wise maps acquired from VBM and SBM, respectively, the parameters were assessed for each of 60 GM regions (Table S1), parcellated as in the Hammers atlas (15), as the average across all voxels within it. Having estimated diffusion tensor-derived parameters, including FA, MD, AD, and RD as voxel-wise maps on the WM tract skeleton acquired from TBSS, the parameters were computed for each of 48 WM tracts (Table S2), parcellated as in the ICBM DTI-81 atlas (16), as the average across all voxels within it. In sum, we considered two parameters of GM and four parameters of WM, which yielded eight combinations of GM and WM parameters. For each combination of GM and WM parameters, parameter values of 60 GM regions and 48 WM tracts composed a total of 108 neuroanatomical features.

### Feature selection by regularized regression

Reducing the number of features is important, especially for data with a large number of features and a limited number of observations. The limited number of observations in relation to the number of features may lead to overfitting to the noise, and regularization is a technique that enables to reduce or prevent overfitting by introducing additional information or constraints on a model. Because all of the 108 features may not include useful and necessary information for classification, we selected a sparse set of features by applying regularized regression. Specifically, the lasso (17) and elastic net (18) were used for regularized logistic regression. The lasso includes a penalty term, or a regularization parameter, λ, that constrains the size of coefficient estimates in a logistic regression model. Because an increase in λ leads to more zero-valued coefficients, the lasso provides a reduced logistic regression model with fewer predictors. The elastic net also produces a reduced logistic regression model by setting coefficients to zero, especially by including a hybrid regularization parameter of the lasso and ridge regression, overcoming the limitation of the lasso in treating highly correlated predictors (19).

For the classification between each pair of the three groups, we applied the lasso and elastic net to identify important predictors among the 108 neuroanatomic features in a logistic regression model. The 108 features of all individuals in each pair of the three groups were standardized to compose a data matrix, **A**, in which each row represented one observation and each column represented one predictor. To correct for effects of individuals' age and sex on the GM and WM parameters, a residual forming matrix, **R**, was generated: **R** = **I**-**C**(**C**^T^**C**)^−1^**C** where **I** was an identity matrix and **C** was a matrix coding confounding covariates of age and sex. It was then applied to **A** to obtain residuals after regressing out the confounding covariates: **X** = **RA**.

Given the adjusted data matrix, **X**, and the response, **Y**, that coded two classes of individuals, 10-fold cross-validation (CV) was used to search for a regularization parameter, λ_MinErr_, that provided the minimum error in terms of deviance, defined as negative log-likelihood for the tested model averaged over the validation folds. Alternatively, because a CV curve has errors at each λ tested, a regularization parameter, λ_1SE_, that was found within one standard error of the minimum CV error in the direction of increasing regularization from λ_MinErr_ was also considered. That is, sparser features were selected at λ_1SE_, whereas sparse features were determined at λ_MinErr_. This procedure for seeking a regularized logistic regression model with fewer predictors was repeated for every combination of GM and WM parameters comprising the 108 neuroanatomical features.

### Performance of selected features

To assess the usefulness of the sparse and sparser features, performance was compared between the model with a reduced number of features and the model with all the 108 features in support vector machines (SVMs) by measuring the receiver operating characteristic (ROC) curve. With a linear kernel as the kernel function and hyperparameters optimized by five-fold CV, an SVM was trained for all individuals in each pair of the three groups. The area under the ROC curve (AUC) was computed for each model as a quantitative measure of its performance. DeLong tests (20) were employed to compare the AUC between each pair of models. When the AUC differed at a *p*-value of 0.05, performance was considered not to be comparable in two models.

### Classification accuracy

**Figure 1:**
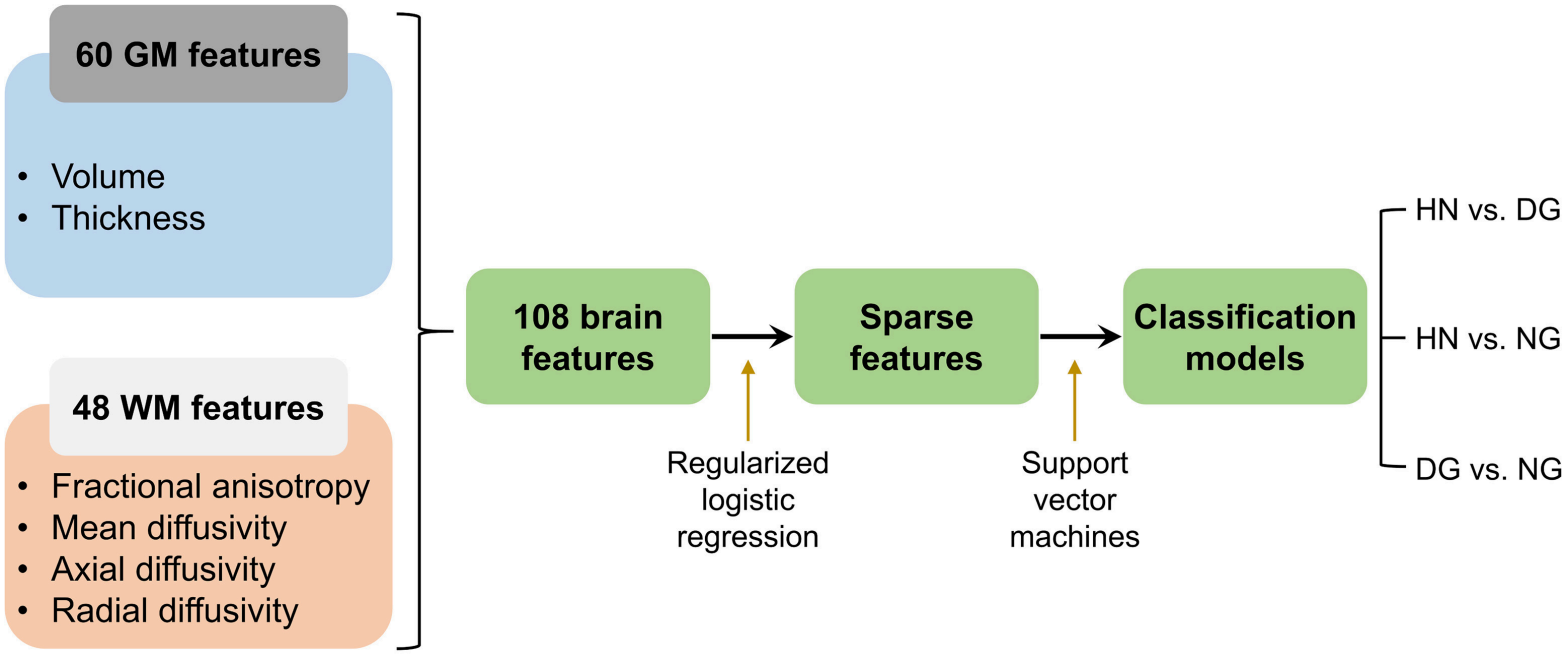
Schematic procedures from the generation and selection of neuroanatomical features to the construction of models for the classification between disordered gamers (DG) and healthy non-gamers (HN), between normal gamers (NG) and HN, and between DG and NG. GM, gray matter; WM, white matter.

Schematic procedures from the generation and selection of features to the construction of classification models is presented in Figure 1. For each pair of the three groups, SVM classification models were generated using the selected features as predictors. We assessed accuracy of the classification models by employing a leave-one-out CV scheme, such that out-of-sample classification accuracy was computed for each left-out individual and then it was averaged across all individuals. The statistical significance of accuracy was estimated by employing permutation tests. An empirical null distribution for classifying between each pair of the three groups was generated by repeatedly permuting the labels of individuals and measuring accuracy associated with the permuted labels. When accuracy measured for the unpermitted labels was higher than or equal to the null distribution at a *p*-value of 0.05, that was determined to be significantly different from the chance level (accuracy = 50%). In addition, a confusion matrix was visualized to describe sensitivity and specificity regarding the distinction between each pair of the three groups.

## Results

### Feature selection

**Figure 2:**
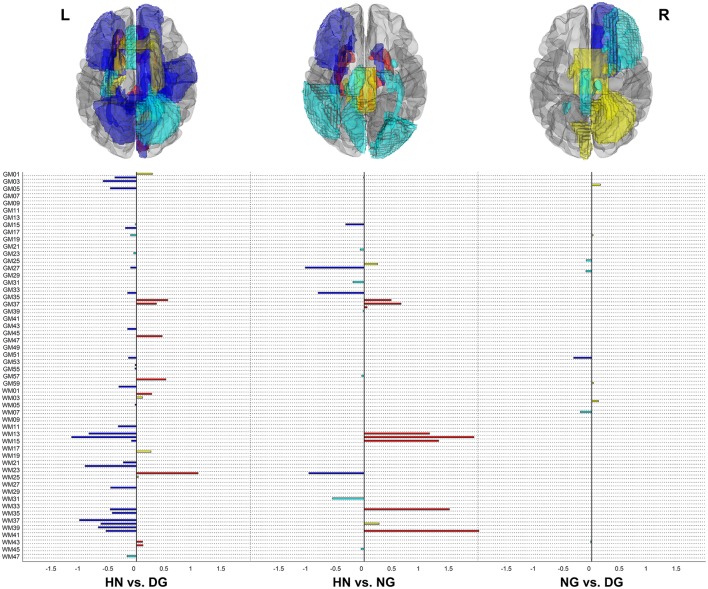
Selected neuroanatomical features in regularized logistic regression for the classification between each pair of three groups. Disordered gamers (DG) were coded as 1 in the classification between healthy non-gamers (HN) and DG, normal gamers (NG) as 1 in the classification between HN and NG, and DG as 1 in the classification between NG and DG. The size of a bar represents the size of the respective feature's coefficient, such that features of non-zero coefficients are selected ones. The rendered brains depict gray matter and white matter components corresponding to the selected features from a superior view. Features in red or blue indicate ones included in sparser features determined at λ_1SE_ as well as in sparse features determined at λ_MinErr_, whereas those in yellow or magenta indicate ones included only in sparse features. The labels of brain components are as provided in Tables S1 and S2. L, left; R, right.

Figure 2 displays selected features among the 108 features with their coefficient estimates, and Table 1 describes related fitting information of the regularized logistic regression model for the classification between each pair of the three groups. In addition, Figure S1 shows which λ yielded the minimum CV error and how many features were selected at λ_1SE_ as well as at λ_MinErr_. The minimum CV error was obtained in feature selection by the lasso (lasso weight = 1) for the classification between the healthy non-gamers and normal gamers and by the elastic net (lasso weight = 0.5) for the other classification.

**Table 1:** Fitting information of regularized logistic regression for the classification between each pair of three groups.

		**HN vs. DG**	**HN vs. NG**	**NG vs. DG**
Parameter	GM	Thickness	Thickness	Volume
	WM	FA	RD	MD
Lasso weight		0.5	1	0.5
Sparse features selected at λ_MinErr_	CV error	37.3681	41.7876	133.3857
	No. of features	43	21	11
Sparser features selected at λ_1SE_	CV error	46.5681	50.0435	141.2622
	No. of features	34	12	1

*The lasso weight indicates whether regularized logistic regression was conducted using the lasso (lasso weight = 1) or elastic net (lasso weight = 0.5)*.

*HN, healthy non-gamers; DG, disordered gamers; NG, normal gamers; GM, gray matter; WM, white matter; FA, fractional anisotropy; RD, radial diffusivity; MD, mean diffusivity; CV, cross-validation*.

In the discrimination of the disordered gamers from the healthy non-gamers, 43 features selected at λ_MinErr_ comprised the thickness of 24 GM regions and the FA of 19 WM tracts, and 34 features selected at λ_1SE_ comprised the thickness of 15 GM regions and the FA of 19 WM tracts. In the distinction of the normal gamers from the healthy non-gamers, 21 features selected at λ_MinErr_ comprised the thickness of 12 GM regions and the RD of 9 WM tracts, and 12 features selected at λ_1SE_ comprised the thickness of 6 GM regions and the RD of 6 WM tracts. In the classification between the disordered and normal gamers, 11 features selected at λ_MinErr_ comprised the volume of 7 GM regions and the MD of 4 WM tracts, and one feature selected at λ_1SE_ corresponded to the volume of one GM region.

### Performance of selected features

**Figure 3:**
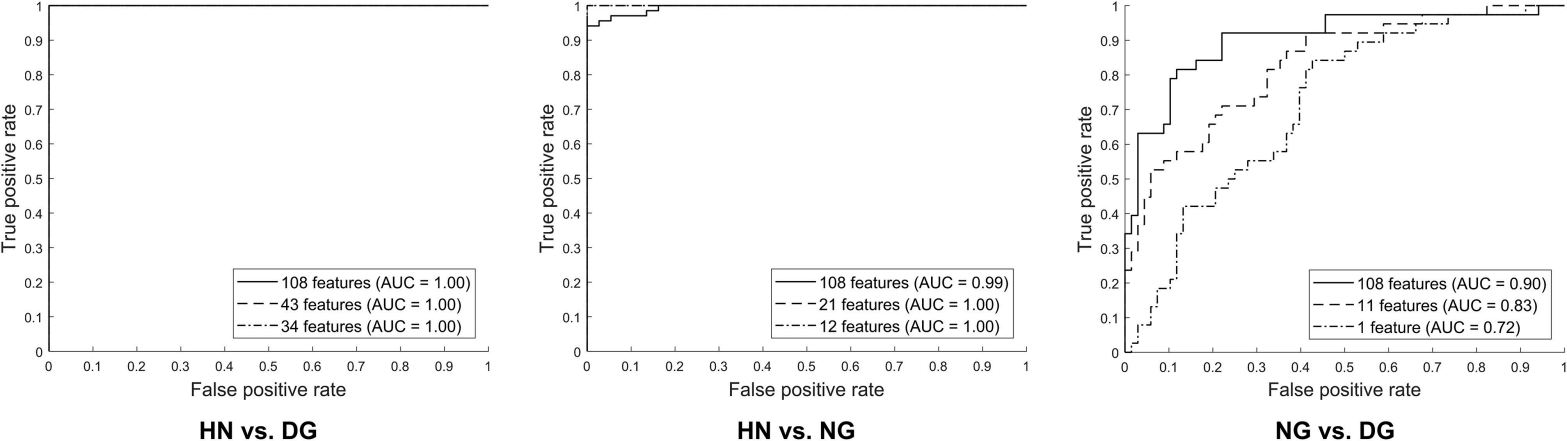
Comparison of performance in terms of the area under the receiver operating characteristic curve (AUC) between models without and with feature selection for the classification between each pair of three groups by support vector machines. The model of 108 features (indicated by solid line) corresponds to that without feature selection, whereas the models of reduced numbers of features correspond to those with sparse and sparser features selected at λ_MinErr_ (indicated by dashed line) and λ_1SE_ (indicated by dash-dot line), respectively. HN, healthy non-gamers; DG, disordered gamers; NG, normal gamers.

Between the model with a reduced number of features and the model with all the 108 features, performance was comparable in terms of the AUC in the discrimination between each type of the gamers and the healthy non-gamers by SVMs (Figure 3). In the classification between the disordered and normal gamers, the model with the features selected either at λ_MinErr_ (AUC = 0.83, *p* = 0.006) or at λ_1SE_ (AUC = 0.72, *p* < 0.001) showed poorer performance than the model with all the 108 features (AUC = 0.90).

### Classification accuracy

**Figure 4:**
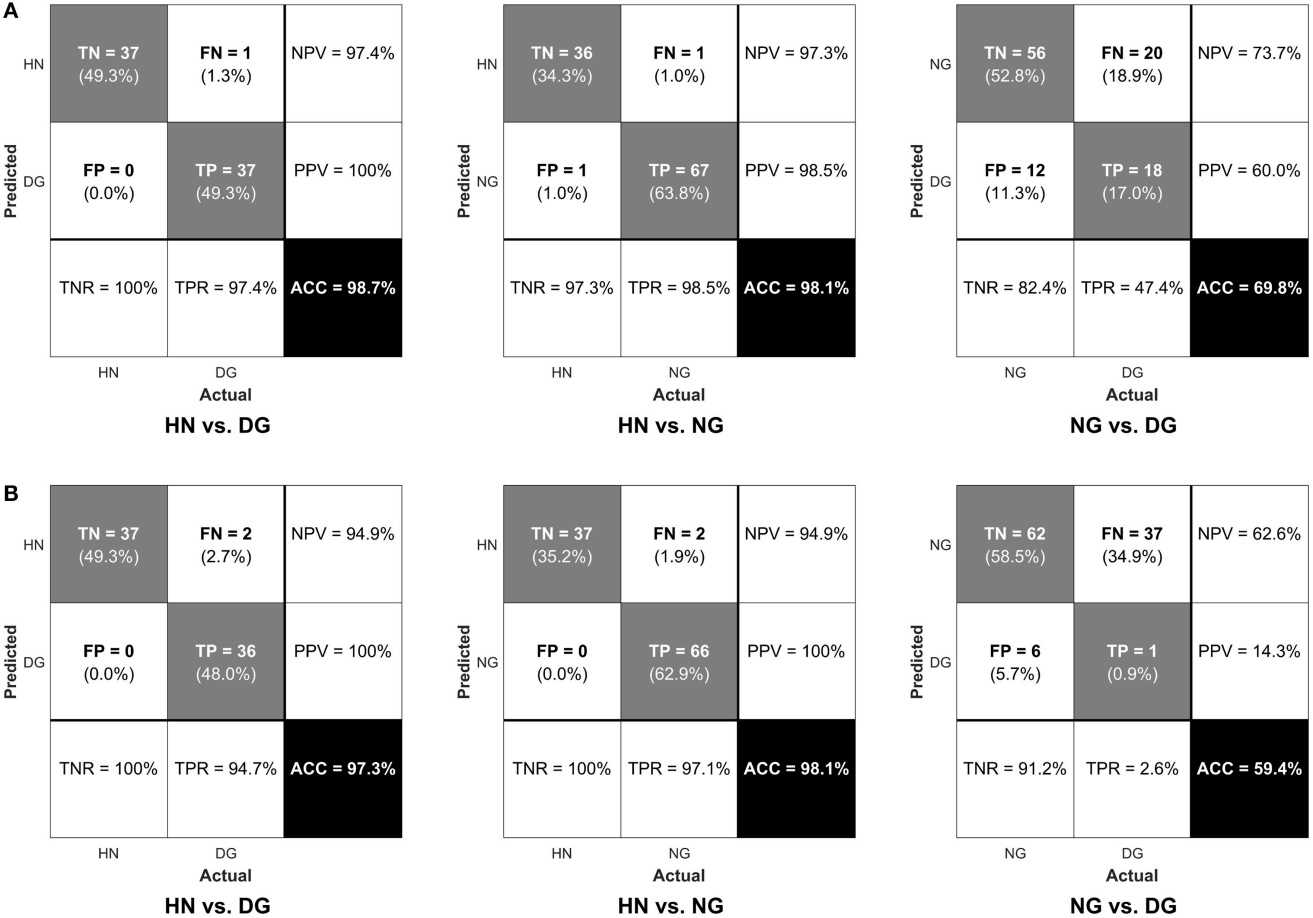
Confusion matrices in the classification between each pair of three groups when using **(A)** sparse and **(B)** sparser features determined at λ_MinErr_ and at λ_1SE_, respectively, in support vector machines. The lower-right cell represents classification accuracy (ACC), the lower-left cell true negative rate (TNR) or specificity, the lower-middle cell true positive rate (TNR) or sensitivity, the upper-right cell negative predictive value (NPV), and the middle-right cell positive predictive value (PPV). TP, true positive; TN, true negative; FP, false positive; FN, false negative.

In classification by SVMs using the features selected at λ_MinErr_, accuracy was greater than 98%, significantly higher than the chance level (*p* < 0.001), in the distinction of each type of the gamers from the healthy non-gamers (Figure 4A). Accuracy was still significantly higher than the chance level (*p* = 0.002) but as low as 69.8% in the classification between the disordered and normal gamers, specifically showing low sensitivity (47.4%) in the correct identification of the disordered gamers. The sparser features determined at λ_1SE_ exhibited similar performance (Figure 4B) but showed much lower sensitivity (2.6%) in the correct distinction of the disordered gamers from the normal gamers.

## Discussion

In this study, we sought to examine whether the pathological and non-pathological gamers as categorized with the IGDS proposed in the DSM-5 could be represented by sparse neuroanatomical features. The disordered and normal gamers were represented in terms of 43 and 21 features, respectively, in relation to the healthy non-gamers. In addition, the disordered gamers were represented in terms of 11 features in relation to the normal gamers. Using the sparse neuroanatomical features, the disordered and normal gamers could be discriminated successfully from the healthy non-gamers, but the classification between the disordered and normal gamers was relatively challenging.

Symptom-based descriptive categorization of IGD with the IGDS proposed in the DSM-5 is being widely adopted. Although empirical validity of the IGDS has been confirmed in multiple countries (3, 21, 22), the threshold of experiencing five or more IGDS items may not be a definite choice, and other ways of categorizing individuals playing Internet-based games may be suggested (12). Since multiple types of clinical data, such as brain imaging data as well as demographic, behavioral, and symptomatic data, become increasingly available, additional data could be preferably employed for the diagnosis of mental illness. In particular, due to the massiveness of quantitative information, brain imaging data are suited for computational approaches and would be useful for prediction. Indeed, brain imaging data have been shown to have superior predictive values compared to other clinical data in prediction for solving a clinically relevant problem (23).

As ML-based diagnostic classification has been recently applied to other addictive behaviors and disorders (24–28), symptom-based categorization of IGD also appears to face a challenge of computation-based classification. Because anatomical abnormalities of the brain following IGD have been repeatedly reported in previous studies (5–7, 9), we considered such neuroanatomical information from brain imaging data potential biomarkers for the diagnosis of IGD. In this study, our goal was to identify a set of important neuroanatomical features that could provide adequately high classification performance, beyond describing neuroanatomical differences between classes of individuals.

We selected important ones, among 108 neuroanatomical features, thorough regularized regression. When we considered eight combinations of GM and WM parameters, different combinations of parameters were selected for distinguishing each pair of the three groups. The combination of the thickness of GM regions and the integrity of WM tracts was better for distinguishing the pathological gamers from the healthy non-gamers, whereas the combination of the volume of GM regions and the diffusivity of WM tracts was better for distinguishing the pathological gamers from the non-pathological gamers. Furthermore, although many brain components commonly served as neuroanatomic features that were important for the distinction of the pathological and non-pathological gamers from the healthy non-gamers, some GM regions and WM tracts characterized the non-pathological gamers, but not the pathological gamers. These findings indicate that there may not be a universally best performing combination of GM and WM parameters as neuroanatomical biomarkers, so that a specific combination of GM and WM parameters needs to be selected according to groups to be classified.

The smaller number of the sparse features for the distinction of the non-pathological gamers compared with the distinction of the pathological gamers, from the healthy non-gamers, reflects that the non-pathological gamers are at a transitional stage between the pathological gamers and healthy non-gamers. In addition, the fewer sparse features for the classification between the two types of the gamers than for the discrimination between each type of the gamers and the healthy non-gamers denotes that the pathological and non-pathological gamers were less dissimilar to each other in terms of neuroanatomy than to them being dissimilar from the healthy non-gamers. Accordingly, the classification models generated with the sparse features yielded accuracy exceeding 98% in the discrimination between each type of the gamers and the healthy non-gamers but accuracy below 70% in the classification between the two types of the gamers. That is, the non-pathological gamers were distinguishable from the healthy non-gamers as well as the pathological gamers were, but there were limitations in distinguishing between the pathological and non-pathological gamers.

This relatively low distinguishability between the two types of the gamers seems to suggest a few notions. Firstly, a mismatch between symptom-based categorization and computation-based classification may be proposed. Although the proposed diagnostic threshold of experiencing five or more criteria in the IGDS was conservatively chosen to prevent the over-diagnosis of IGD (12), the presence of gamers suffering considerable pathological changes in neuroanatomy but not satisfying the IGD threshold may not be disregarded. In particular, we only included gamers who satisfied IGDS items much lower than the IGD threshold as the normal gamers, so that gamers diagnosed as not having IGD could be generally further away from non-gaming healthy individuals than shown in this study. Secondly, a challenge in classification relying only on neuroanatomical biomarkers may be noted. Classification performance could be improved by including other biomarkers that can capture greater dissimilarity between the pathological and non-pathological gamers. In particular, because functional changes in the brain are also demonstrated in IGD (29–33), function as well as anatomy of the brain could be considered brain biomarkers. In addition, we want to note that changes in the brain only constitute part of the multidimensional facets of Internet gaming addiction, so that other factors, not least various internal and external risk factors for Internet gaming addiction (34), should be included in more complete models for the classification between pathological and non-pathological gamers as well as the distinction of gamers from non-gaming healthy individuals.

Here, we have employed regularized regression, using sparsity-promoting estimators such as the lasso and elastic net, to identify important features for classification models. There are actually methodological variations in feature selection or dimensionality reduction, and a variety of approaches may be employed for the use of selected features in model construction (35). Our approach using regularized regression entails a priori assumption concerning sparsity in neuroanatomical features. Provided that such an assumption is acceptable, as we believed in this study, regularized regression could be a plausible approach, and the selected set of sparse features would be expected to compose classification models of adequately high performance. But it is notable that simpler classification models based on greater sparsity may not always exhibit comparable or improved performance. Indeed, among different choices of the degree of sparsity according to a regularization parameter, greater sparsity was not likely to provide a better performing model specifically in more challenging classification problems, such as the classification between the pathological and non-pathological gamers.

In addition, we have used SVMs as an ML technique for constructing classification models, because they are among most popular ones. Other advanced methods may be used to improve classification performance, although comparative performance between different methods may not be concluded because of the dependence of performance on experimental scenarios (19). On the other hand, for comparative performance between classical statistical methods and ML techniques, we conducted classification by logistic regression as well and showed that the two methods, namely logistic regression and SVMs, were comparable in the performance of classification (Figure S2). It may be iterated that classical statistical methods are not always inferior to ML techniques in classification performance (36).

In the current study, we have revealed that symptom-based categorization of IGD could be represented in terms of sparse neuroanatomical biomarkers that composed classification models. Furthermore, we have demonstrated that non-pathological gamers could be less distinguishable from pathological gamers than from non-gaming healthy individuals, in terms of neuroanatomy. We thus suggest that although current diagnostic systems rely on descriptive categorization such as the DSM-5 as the gold standards, non-pathological gamers may need to be diagnosed with more care by employing objective biomarkers such as those associated with neuroanatomical alterations. Adoption of computational approaches seems to be an irreversible trend in psychiatry, but there may be a long way to go to practically apply those to clinical environments. Search for the optimal selection of sparse features from brain imaging and other clinical data needs to be conducted in subsequent studies, and in the long term, these efforts would promote the computation-based diagnosis of IGD.

## Author contributions

D-JK and J-WC were responsible for the study concept and design. HC conducted the clinical characterization and selection of participants. CP analyzed the data and drafted the manuscript. All authors critically reviewed content and approved final version for publication.

### Conflict of interest statement

The authors declare that the research was conducted in the absence of any commercial or financial relationships that could be construed as a potential conflict of interest.
