# Supplementary material for: Discriminating Pathological and Non-pathological Internet Gamers Using Sparse Neuroanatomical Features
Source: Front Psychiatry. 2018 Jun 29;9:291. doi: 10.3389/fpsyt.2018.00291 (PMC6033968; doi:10.3389/fpsyt.2018.00291)
Supplement: Supplementary file 1 [file Data_Sheet_1.pdf]

Supplementary Material

Discriminating Pathological and Non-pathological Internet Gamers Using Sparse Neuroanatomical Features

Chang-hyun Park, Ji-Won Chun, Hyun Cho, Dai-Jin Kim*

*** Correspondence:** Dai-Jin Kim: kdj922@catholic.ac.kr

1 Supplementary Figures and Tables

1.1 Supplementary Figures

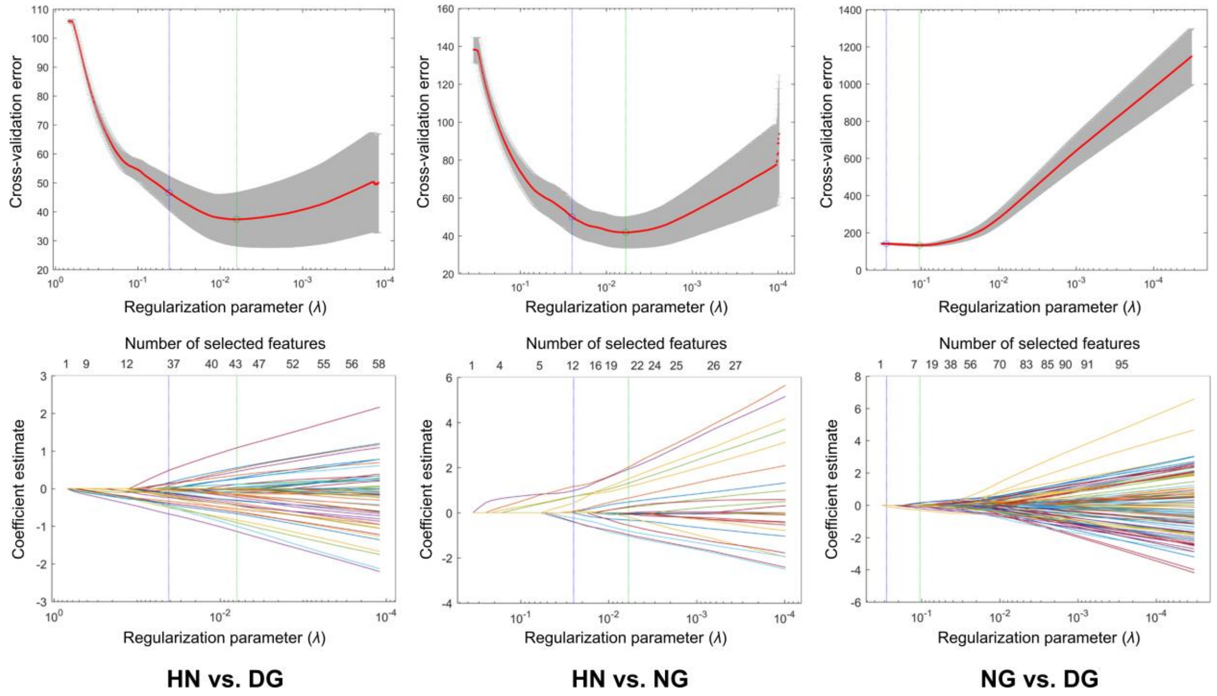

Figure S1. Trace plots of cross-validation (CV) error across a range of values of the regularization parameter (λ) (upper panels) and respective coefficients fit by the lasso or elastic net (lower panels) in regularized logistic regression. The plots are depicted in logarithmic scaling. The green circle and dashed line indicate the regularization parameter, λ_{MinErr}, with minimum CV error, and the blue circle and dashed line indicate the alternative regularization parameter, λ_{1SE}, located within one standard error of the minimum CV error in the direction of increasing regularization from λ_{MinErr}. In upper panels, points in red and error bars in gray represent the mean and standard deviation, respectively, of CV error. HN, healthy non-gamers; DG, disordered gamers; NG, normal gamers.

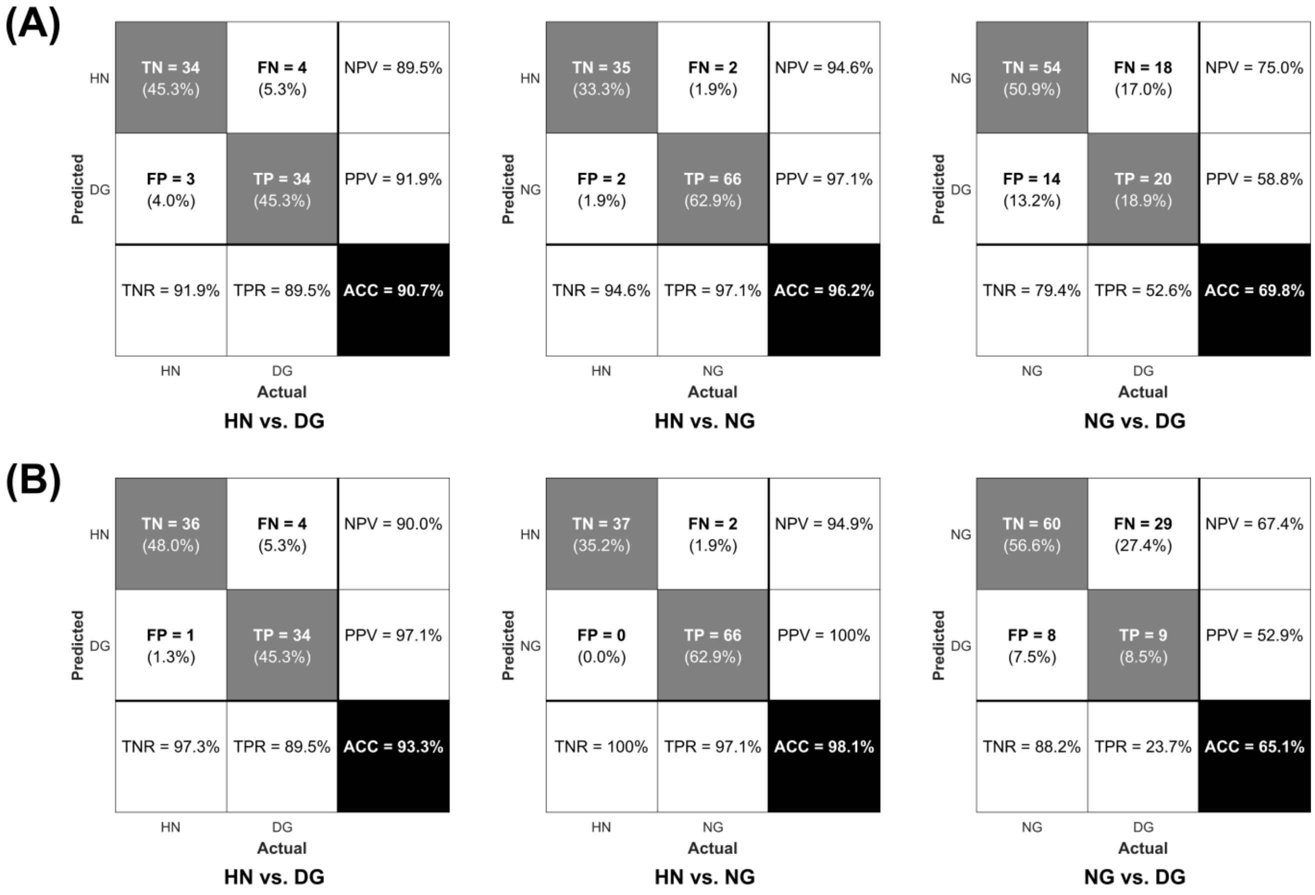

Figure S2. Confusion matrices in the classification between each pair of three groups when using (A) sparse and (B) sparser features determined at $\lambda_{\mathrm{MinErr}}$ and λ_{1SE}, respectively, in logistic regression. The lower-right cell represents classification accuracy (ACC), the lower-left cell true negative rate (TNR) or specificity, the lower-middle cell true positive rate (TNR) or sensitivity, the upper-right cell negative predictive value (NPV), and the middle-right cell positive predictive value (PPV). TP, true positive; TN, true negative; FP, false positive; FN, false negative.

1.2 Supplementary Tables

Table S1. Sixty gray matter (GM) regions for which volume and thickness were assessed as neuroanatomical features.

Label	Name
GM01	Hippocampus L
GM02	Hippocampus R
GM03	Amygdala L
GM04	Amygdala R
GM05	Anterior medial temporal lobe L
GM06	Anterior medial temporal lobe R
GM07	Anterior lateral temporal lobe L
GM08	Anterior lateral temporal lobe R
GM09	Ambient and parahippocampus gyri L

GM10	Ambient and parahippocampus gyri R
GM11	Superior temporal gyrus L
GM12	Superior temporal gyrus R
GM13	Inferior middle temporal gyrus L
GM14	Inferior middle temporal gyrus R
GM15	Fusiform gyrus L
GM16	Fusiform gyrus R
GM17	Cerebellum L
GM18	Cerebellum R
GM19	Insula L
GM20	Insula R
GM21	Lateral occipital lobe L
GM22	Lateral occipital lobe R
GM23	Anterior cingulate gyrus L
GM24	Anterior cingulate gyrus R
GM25	Posterior cingulate gyrus L
GM26	Posterior cingulate gyrus R
GM27	Middle frontal gyrus L
GM28	Middle frontal gyrus R
GM29	Posterior temporal lobe L
GM30	Posterior temporal lobe R
GM31	Inferior lateral parietal lobe L
GM32	Inferior lateral parietal lobe R
GM33	Caudate nucleus L
GM34	Caudate nucleus R
GM35	Accumbens nucleus L
GM36	Accumbens nucleus R
GM37	Putamen L
GM38	Putamen R
GM39	Thalamus L
GM40	Thalamus R
GM41	Pallidum L
GM42	Pallidum R
GM43	Precentral gyrus L
GM44	Precentral gyrus R
GM45	Gyrus rectus L
GM46	Gyrus rectus R
GM47	Orbito-frontal gyrus L
GM48	Orbito-frontal gyrus R
GM49	Inferior frontal gyrus L
GM50	Inferior frontal gyrus R
GM51	Superior frontal gyrus L
GM52	Superior frontal gyrus R
GM53	Postcentral gyrus L

GM54	Postcentral gyrus R
GM55	Superior parietal gyrus L
GM56	Superior parietal gyrus R
GM57	Lingual gyrus L
GM58	Lingual gyrus R
GM59	Cuneus L
GM60	Cuneus R

L, left; R, right.

Table S2. Forty-eight white matter (WM) tracts for which fractional anisotropy, mean diffusivity, axial diffusivity, and radial diffusivity were assessed as neuroanatomical parameters.

Label	Name
WM01	Middle cerebellar peduncle
WM02	Pontine crossing tract
WM03	Genu of corpus callosum
WM04	Body of corpus callosum
WM05	Splenium of corpus callosum
WM06	Fornix
WM07	Corticospinal tract R
WM08	Corticospinal tract L
WM09	Medial lemniscus R
WM10	Medial lemniscus L
WM11	Inferior cerebellar peduncle R
WM12	Inferior cerebellar peduncle L
WM13	Superior cerebellar peduncle R
WM14	Superior cerebellar peduncle L
WM15	Cerebral peduncle R
WM16	Cerebral peduncle L
WM17	Anterior limb of internal capsule R
WM18	Anterior limb of internal capsule L
WM19	Posterior limb of internal capsule R
WM20	Posterior limb of internal capsule L
WM21	Retrolenticular part of internal capsule R
WM22	Retrolenticular part of internal capsule L
WM23	Anterior corona radiata R
WM24	Anterior corona radiata L
WM25	Superior corona radiata R
WM26	Superior corona radiata L
WM27	Posterior corona radiata R
WM28	Posterior corona radiata L
WM29	Posterior thalamic radiation R
WM30	Posterior thalamic radiation L

WM31	Sagittal stratum R
WM32	Sagittal stratum L
WM33	External capsule R
WM34	External capsule L
WM35	Cingulum R
WM36	Cingulum L
WM37	Cingulum R
WM38	Cingulum L
WM39	Fornix / Stria terminalis R
WM40	Fornix / Stria terminalis L
WM41	Superior longitudinal fasciculus R
WM42	Superior longitudinal fasciculus L
WM43	Superior fronto-occipital fasciculus R
WM44	Superior fronto-occipital fasciculus L
WM45	Uncinate fasciculus R
WM46	Uncinate fasciculus L
WM47	Tapetum R
WM48	Tapetum L

L, left; R, right.
